# Supplementary material for: Findings of a feasibility study of pre-operative pulmonary rehabilitation to reduce post-operative pulmonary complications in people with chronic obstructive pulmonary disease scheduled for major abdominal surgery
Source: F1000Res. 2020 Mar 9;9:172. [Version 1] doi: 10.12688/f1000research.22040.1 (PMC7702168; doi:10.12688/f1000research.22040.1)
Supplement: Peer review history

# Peer review: Reviewer response for version 1

**DOI:** 10.5256/f1000research.24306.r61114

**Published:** 2020-11-26

**Authors:** Gary Minto

**Affiliations:** 1 Department of Anaesthesia, Derriford Hospital, Plymouth Hospitals NHS Trust, Plymouth, UK

Feasibility of an RCT of pulmonary rehab for 3 weeks vs standard of care in COPD patients before major surgery.

 Really well written - clear statements of the scale of the problem and clear statements of the need for evidence. Clear rationale for plausibility of intervention. Clear focus on the purpose of THIS study - is pre-operative pulmonary rehabilitation feasible? (rather than does it work).

 Decision-making points about whether to proceed to a “full” study were defined and the feasibility endpoints were well designed.

 Even the methodology BEFORE feasibility acknowledges a likely problem - authors were assuming a drop out of 1/3 of pts who had been recruited! - “this 48 dataset target, based on known drop-out rates from pulmonary rehabilitation and potential further data loss due to surgical scheduling, we anticipated that we would need to recruit 72 patients”.

 Points of the pathway to identify potentially eligible participants comprehensively explored - Only 3 randomised! Thorough evaluation of why - i.e. issues identified related to the confirmation of diagnosis of COPD within a pathway towards major body cavity surgery. Not only in the 2 participating centres but also in 3 further centres (in detail) plus findings from a UK national survey of preop units.

**IMPLICATIONS for future research:**

 Agree wholeheartedly with the authors' conclusion that under current UK surgical pathway conditions, an RCT of pulmonary rehab as prehabilitation for COPD before major surgery is not feasible. There is a commendable readiness on the part of the investigators to change their outlook in the face of this feasibility study evidence.

 Why is it that such a large proportion of the patients who WERE identified as having COPD and were eligible (n = 45 , figure 2), declined to participate - often on the basis that they had “too many appointments/too much to think about/too far to travel? As the authors put it: “patients often do not want to engage with pulmonary rehabilitation at a time when they are dealing with a new, life changing diagnosis and having burdensome, potentially toxic cancer treatment".

 The authors make a key point that when pulmonary rehab is framed as an “optional” research extra, then it seems acceptable to opt out. I concur with their suggestion that pulmonary rehab could instead be framed (in those hospitals which have the resource) as an important part of the surgical pathway; and that future research into effectiveness might be better conducted by comparing clinical outcomes after surgery in hospitals with or without “Surgery School” (in a manner akin to research into the effectiveness of Enhanced Recovery Pathways).

**IMPLICATIONS for practice:**

 It is striking that pulmonary rehabilitation is a proven and effective therapy (with a great benefit in terms of QALYs) which is meant to be offered to ALL patients with a diagnosis of COPD, yet so few patients with COPD (300 participants per year being assessed for major surgery in each of the 2 sites are estimate to have the condition) arrived at the preop clinic having already had experience of pulmonary rehab. 

 So wider questions - implied by the authors in the discussion, but perhaps worth stating explicitly are things like:

- Why are so many people with COPD undiagnosed?
- Why do many of those *with *a diagnosis still have no personal experience of pulmonary rehabilitation?
- Why are we having to use attendance at hospital for assessment for surgery as an opportunistic moment to introduce pulmonary rehabilitation?

These might be addressed by better education of the general population and health care professionals. 

 A philosophical question: Is it ethical to proceed to perform major surgery when patients have opted out of engaging with prehabilitation? 

 In the current scenario there is (very) limited clear cut evidence that pulmonary rehabilitation has utility and might improve clinical outcomes after surgery? It appears that an RCT of this therapy isn't possible in the UK without a radical overhaul of surgical pathways. How should we now proceed to find this evidence (whichever way the evidence pans out)?

**Minor issues:**

 Please make sure the pulmonary rehab program is clearly defined. In the introduction - pulmonary rehab is explained as 1 hour of exercise and 1 hour of education twice a week for 6 weeks. - What is the format of education delivered for 2 hours a week for 6 weeks?

 Attendance - the logistics of getting to classes appears to be a problem or possibly a factor in refusals. Could classes be virtual?

 Figure 2 - typo in box 3 left hand side - should read 45 were approached (not 65).

 Page 4 top line - is this a typo? exclusions: “Patients scheduled cardiac, thoracic and orthopaedic surgery and orthopaedic surgery"

 Page 8 typo? "COPD may not have been formally diagnosed (UNTIL) after the pre-assessment clinic".

Is the work clearly and accurately presented and does it cite the current literature?

Yes

If applicable, is the statistical analysis and its interpretation appropriate?

Not applicable

Are all the source data underlying the results available to ensure full reproducibility?

Yes

Is the study design appropriate and is the work technically sound?

Yes

Are the conclusions drawn adequately supported by the results?

Yes

Are sufficient details of methods and analysis provided to allow replication by others?

Yes

Reviewer Expertise:

perioperative medicine

I confirm that I have read this submission and believe that I have an appropriate level of expertise to confirm that it is of an acceptable scientific standard.

# Peer review: Reviewer response for version 1

**DOI:** 10.5256/f1000research.24306.r71179

**Published:** 2020-09-30

**Authors:** Ianthe Boden

**Affiliations:** 1 Department of Physiotherapy, Launceston General Hospital, Launceston, Tas, Australia; 2 University of Melbourne, Melbourne, Vic, Australia

This is a well-conceived and beautifully reported feasibility study that provides vital information required by clinical trialists and grant funding bodies to consider the design of future research in the field of pre-operative optimisation in this high-risk surgical cohort.

 A preexisting diagnosis of COPD significantly increases a person's risk of suffering a complication after major surgery. There is preliminary evidence that for patients with COPD awaiting thoracic surgery that pulmonary rehabilitation (a supervised twice-weekly program of tailored education and physical exercise) in the weeks immediately prior to surgery may reduce the risk of postoperative complications and improve recovery after surgery. Pulmonary rehabilitation is an established clinical program within developed countries. There is definitive evidence of the benefit and cost-effectiveness of pulmonary rehabilitation as a primary therapy to improve symptoms and quality of life for people with stable COPD. However, it is yet to be established as an effective program to preoptimise patients with COPD awaiting major abdominal surgery. This is an intriguing possibility.

 This paper reports the results of a multicentre randomised controlled trial that assessed the feasibility of conducting a trial of preoperative pulmonary rehabilitation for patients with COPD and awaiting major abdominal surgery. This trial was pre-registered with feasibility outcomes specified a priori.

 Despite a strong research group with significant experience and track record, two well-resourced centers of excellence as investigating sites, and a well concieved protocol to provide evidence based preoperative pulmonary rehabilitation, this trial was found to be unfeasible due to exceptionally low recruitment uptake.

 Despite all their best efforts (free transport to pulmonary rehabilitation and flexible appointment times) the reasons were reported as being predominately outside the control of researchers; namely established presurgical pathways and finite time scale from time of listing for surgery to date of surgery often being shorter (2 - 3 weeks) than the time period allowable of being able to provide an effective dose of preoperative pulmonary rehabilitation (4 - 6 weeks).

 However, I was unable to determine the exact proportions of the various reasons for eligible patients to not be recruited into the trial as the numbers in the flow chart in the box of 'Declined' patients do not add up to the total stated of n=42. I would also suggest that the 'not approached' group are actually ineligible/not suitable as they are not listed for surgery so no longer are eligible criteria. The flow chart's numbers are all a bit off with boxes following the arrows of flow not adding up to the box prior to it in flow order. This requires clarification.

 A few other comments that could be considered within reporting of this paper:

1. It was unclear how a diagnosis of COPD was determined in the eligibility criteria. GOLD standard? Spirometry? Medical record report/coding?
2. The discussion considers that to overcome the barriers identified that the pre-surgical pathway would need to be altered to ensure that a COPD morbidity is recognised earlier and these patients are fed into a pulmonary rehab program at a much earlier time frame than the preoperative anaesthetic assessment clinic.
  I would be interested to hear from the authors what interventions have been recently tested within the CURRENT surgical pathways that prevent postoperative complications e.g preoperative physiotherapy to teach patients breathing exercises inserted into preanaesthetic clinics (Boden *et al.*) or inspiratory muscle training (Kendall *et al.*), and how these could be considered within this specific surgical cohort of patients with COPD. Should more be done to implement these findings within existing pathways? Or should energy be spent in changing the pathway to get more patients into prehab/pulmonary rehabilitation?
3. Also, what reflections do the authors have of their findings in contrast to the emerging body of evidence for 'pre-habilitation' in a general surgical cohort (not refined to COPD)? Recent Spanish (Barabaran Garcia *et al.*) and Canadian (Carli *et al.*) studies did not appear to have limitations to recruiting to a high-intensity preoperative exercise program within 4-6 weeks of surgery. Is this a factor related to the cohort of patients with COPD or related to the different countries? Or something else? A UK trial of prehab for vascular surgery patients also did not have issues with recruitment. I think it would be wise for the authors to discuss these differences in trial conduct. 

The authors should be congratulated on ensuring that this data is available and published in the interests of transparent reporting. It is often disheartening to report null results or trial conduct 'failures'. However, in the interests of future efficient research activities, the paper published here presents excellent information and thought-provoking concepts that challenge our thinking about how, when, and what to do with patients prior to surgery to improve their postoperative outcome. Thankfully the authors did not bury their data along with their disappointment when their hypothesis was unable to be tested to its fullest. This paper is an excellent point of learning for all of us working in the perioperative medicine sector. Thank you.

Is the work clearly and accurately presented and does it cite the current literature?

Partly

If applicable, is the statistical analysis and its interpretation appropriate?

Yes

Are all the source data underlying the results available to ensure full reproducibility?

Partly

Is the study design appropriate and is the work technically sound?

Yes

Are the conclusions drawn adequately supported by the results?

Yes

Are sufficient details of methods and analysis provided to allow replication by others?

Partly

Reviewer Expertise:

Physiotherapy, critical care, perioperative medicine, health economics, clinical trials, postsurgical recovery

I confirm that I have read this submission and believe that I have an appropriate level of expertise to confirm that it is of an acceptable scientific standard.
